# Seeding biosensor cell line that reproduces the Alzheimer tau fold

**DOI:** 10.1016/j.jbc.2025.110952

**Published:** 2025-11-17

**Authors:** Taxiarchis Katsinelos, Sofia Lövestam, Chao Qi, Benjamin Ryskeldi-Falcon, Jennifer A. Macdonald, Gabriel Stephenson, Bernardino Ghetti, Ann McKee, Sjors H.W. Scheres, Michel Goedert

**Affiliations:** 1 MRC Laboratory of Molecular Biology, Cambridge, UK; 2 Department of Pathology and Laboratory Medicine, Indiana University School of Medicine, Indianapolis, Indiana, USA; 3 Alzheimer's Disease and Chronic Traumatic Encephalopathy Research Center, Boston University School of Medicine, Boston, Massachusetts, USA

**Keywords:** neurodegenerative diseases, seeded aggregation, tau filament assembly, Alzheimer's tau fold, biosensor cell line, cryo-EM

## Abstract

The assembly of tau protein into amyloid filaments through templated seeding is believed to underlie the propagation of pathology in neurodegenerative diseases, such as Alzheimer’s disease (AD) and other tauopathies. A commonly used model system for studying this process is through the induction of tau filament formation in cultured cells following the addition of tau seeds isolated from the human brain. However, little is known about the structures of seeded filaments; some biosensor cell lines are unable to reproduce the tau filament structures from AD, because they overexpress tau fragments that do not cover the whole of the ordered filament core. Here, we describe a novel tau seeding biosensor model in human embryonic kidney 293T cells that overexpress residues K297–E391 of human 4R tau. The construct contains an N-terminal hemagglutinin tag, which allows the specific detection of the amplified template. The biosensor cells detected filaments seeded by material from sporadic three-repeat (3R) + four-repeat (4R) tauopathies, with little activity by seeds from 3R-only or 4R-only tauopathies. The sensitivity of seed detection from 3R + 4R tauopathies in our system was similar to or higher than for previously reported biosensors. We also structurally characterized the AD-seeded tau filaments by cryo-EM. Most of the cell-derived filaments consisted of two protofilaments with the Alzheimer's fold but with a “head-to-head” interprotofilament packing. Our results establish a sensitive biosensor cell line with specificity toward seeds from 3R + 4R tauopathies.

The formation of hyperphosphorylated and abnormally phosphorylated filamentous tau inclusions is a major pathological feature of several neurodegenerative diseases, collectively termed tauopathies (1). Alzheimer’s disease (AD) is the most common tauopathy. Disease-causing mutations in *MAPT*, the gene encoding tau protein, together with the invariable presence of filamentous tau inclusions in these cases of frontotemporal dementias, indicate that the assembly of tau into filaments is sufficient to cause neurodegeneration (2, 3).

In the adult human brain, alternative mRNA splicing leads to the expression of six tau isoforms from a single *MAPT* gene (4). Three isoforms have four microtubule binding repeats (4R), whereas the exclusion of exon 10, which encodes the second repeat, leads to the production of three isoforms with three repeats (3R) (5). Tauopathies can be classified according to the isoform composition of tau inclusions as 3R-only, 4R-only, and 3R + 4R tauopathies. In the brains of individuals with AD, a 3R + 4R tauopathy, two types of filaments are present: abundant paired helical filaments (PHFs) and rarer straight filaments (SFs) (6). Cryo-EM work has shown that both filament types consist of two identical C-shaped protofilaments, with distinct interprotofilament packings (ultrastructural polymorphs) (7, 8). The C-shaped core of each protofilament, that is, the Alzheimer's tau fold, comprises amino acids V306–F378 (based on the numbering of the longest 441-amino acid tau isoform). Weaker densities are also visible for amino acid regions 273 to 274, 304 to 305, and 379 to 380. Tau filaments from the brains of individuals with chronic traumatic encephalopathy (CTE) also consist of two types of filaments composed of two identical protofilaments that are linked in different ways; they are similar, but not identical, to the Alzheimer's tau fold, from which they also differ by the presence of a nonproteinaceous density that is enclosed in a hydrophobic cavity of the β-helix region (9). In both cases, the first amino acid of the ordered core (V306) is also the first amino acid of R3, meaning that 3R and 4R tau isoforms can be incorporated indiscriminately, explaining the presence of all six brain tau isoforms in AD and CTE filaments (10).

Aggregated tau species appear sequentially in different regions of the brain during disease progression (11). Stereotypic patterns *via* connected neuronal pathways (12) probably involve the cell-to-cell spread of filamentous tau assemblies (13). Therefore, the conversion of monomeric tau into filamentous species *via* templated seeding, similar to mechanisms in prion diseases, is believed to be a major determinant for the progression of tauopathies (14). Even though the tau species responsible for this process in human brains are unknown, evidence from transgenic mouse models overexpressing mutant human P301S tau suggests that short tau filaments are the major seed-competent species (15).

Cellular biosensors allow researchers to identify and quantify specific biological reactions inside cells (16, 17). In the context of cellular biosensors for the seeded aggregation of tau, the most commonly used systems overexpress fluorescently tagged full-length or truncated tau constructs, which often also carry mutations associated with frontotemporal dementias (18, 19). When these cells take up small amounts of tau amyloid filaments, seeded aggregation of the overexpressed tau leads to the formation of fluorescent puncta that can be quantified and used as a measure of seeded aggregation. Sensitive and relevant cellular biosensors can be used for the detection of tau seeds, the study of disease mechanisms, and the screening of drugs that target seeded tau aggregation (10).

Seeded aggregation should be able to amplify filaments from small amounts of seeds, and the seeded filaments should have the same atomic structures as the seed filaments. The first requirement has been met in several tau biosensor systems, where small amounts of tau seeds were added in the presence of cationic lipids, which led to robust seeded aggregation (18, 19, 20, 21). However, only certain aspects of structural fidelity in seeded tau filaments have been reproduced in cellular systems, such as the partial inheritance of tau amyloid structures (22) and the tau isoform– or mutation-based preferential seeding behaviors (23, 24). Some of these cell lines are incapable of replicating the tau amyloid structures from tauopathies because they overexpress tau fragments that are too short to cover the whole of the ordered core (18, 25).

Here, we describe a tau seeding biosensor model in human embryonic kidney 293T (HEK293T) cells that stably overexpresses a hemagglutinin (HA)-tagged region of 4R human tau spanning residues K297–E391 (HA-tau297–391). The same tau fragment, also called dGAE (26), assembles *in vitro* into PHFs identical to those found in AD brains (27). Our biosensor cells specifically detect filaments with the structures of seeds from 3R + 4R tauopathies, with little activity of seeds from 3R-only or 4R-only tauopathies. The sensitivity of detection for seeds from 3R + 4R tauopathies is similar to or higher than for previously described biosensors. We also show by cryo-EM that seeded aggregation with AD-tau seeds leads to amyloid filaments with two protofilaments with the Alzheimer's amyloid fold but with a “head-to-head” interprotofilament packing. Our results establish a sensitive tau seeding biosensor system with specificity toward seeds from 3R + 4R tauopathies as well as structural fidelity of seeded AD aggregates at the protofilament level.

## Results

### HA-tau297–391 tau biosensor cell line

**Figure 1:**
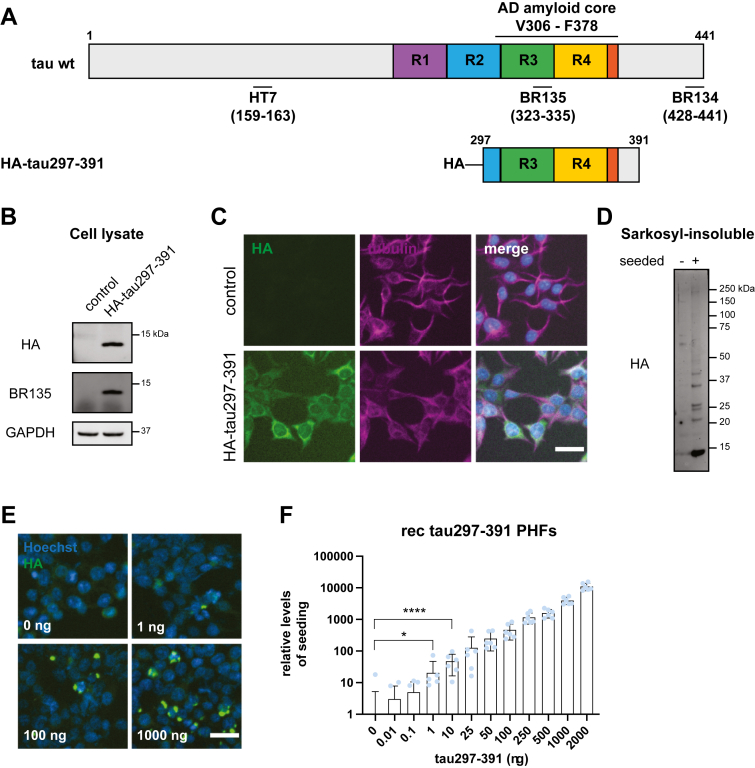
**HA-tau297–391 seeding biosensor cell line.***A*, schematic of full-length 0N4R tau and HA-tagged tau297–391. The epitopes of antitau antibodies HT7, BR135, and BR134 as well as the region of the AD amyloid tau core (V306–F378) are indicated. *B*, Western blot analysis of HA-tau297–391 that was stably overexpressed in HEK293T cells. Total cell lysates were immunoblotted using anti-HA, BR135, and GAPDH antibodies. *C*, immunofluorescence analysis of HA-tau297–391 stably overexpressed in HEK293T cells. Anti-HA and tubulin antibodies were used, whereas nuclei were stained with Hoechst dye. *D*, sarkosyl-insoluble fractions from HA-tau297–391 biosensors unseeded and seeded with 750 ng recombinant PHFs were immunoblotted using the anti-HA antibody. *E*, representative immunofluorescence images from HA-tau297–391 biosensors treated with increasing amounts of recombinant PHFs. *F*, quantification of HA-positive intracellular inclusions in HA-tau297–391 biosensors after seeding with increasing amounts of recombinant PHFs. Image analysis included at least 15,000 cells per condition from three experimental replicates. ∗*p* < 0.05, ∗∗∗∗*p* < 0.0001 by one-way ANOVA with Tukey’s correction, and error bars denote standard deviations. The scale bars (in *C* and *E*) represent 35 μm. AD, Alzheimer’s disease; HA, hemagglutinin; HEK293T, human embryonic kidney 293T cell line; PHF, paired helical filament.

Using lentiviral transduction, we generated stable HEK293T cell lines that overexpress tau297–391. The genome-integrated construct also included an N-terminal human influenza HA tag to distinguish between the expressed protein and the added seeds (Fig. 1*A*). We first explored the expression of HA-tau297–391 by Western blot and immunofluorescence analyses (Fig. 1, *B* and *C*). HA-tau297–391 ran at the predicted molecular weight on SDS-PAGE and displayed a diffuse cytoplasmic distribution by immunohistochemistry. However, when the cells were seeded for 48 h with assembled recombinant tau297–391 PHFs in the presence of Lipofectamine, we detected a large amount of sarkosyl-insoluble HA-tagged material (Fig. 1*D*). Staining of fixed seeded cells identified a dose-dependent increase of HA-positive intracellular foci after treatment with increasing amounts of recombinant tau297–391 PHFs (Fig. 1*E*). To quantify this phenotype, we used automated image analysis in Fiji (28) for the segmentation and detection of individual nuclei and HA-labeled aggregated puncta (Fig. S1*A*). Statistically significant levels of seeding (as compared with a PBS-treated control) were detected at 1 ng (equivalent to approximately 1 nM final concentration) of recombinant PHFs. In contrast, filaments that were assembled from recombinant tau297–391 bearing the frontotemporal dementia–associated mutation P301S did not induce seeding, even at high concentrations (Fig. S1, *B* and *C*). In a control experiment to confirm their seeding competence, the same recombinant tau297–391 P301S filaments displayed robust seeding when added to previously characterized tau RD P301S FRET seeding biosensor cells overexpressing human tau with the P301S mutation (18, 19) (Fig. S1*D*). These data demonstrate a sensitive and quantitative tau seeding assay that responds to the presence of recombinant PHFs.

### Tau seeds extracted from human tauopathies and a transgenic mouse model

**Figure 2:**
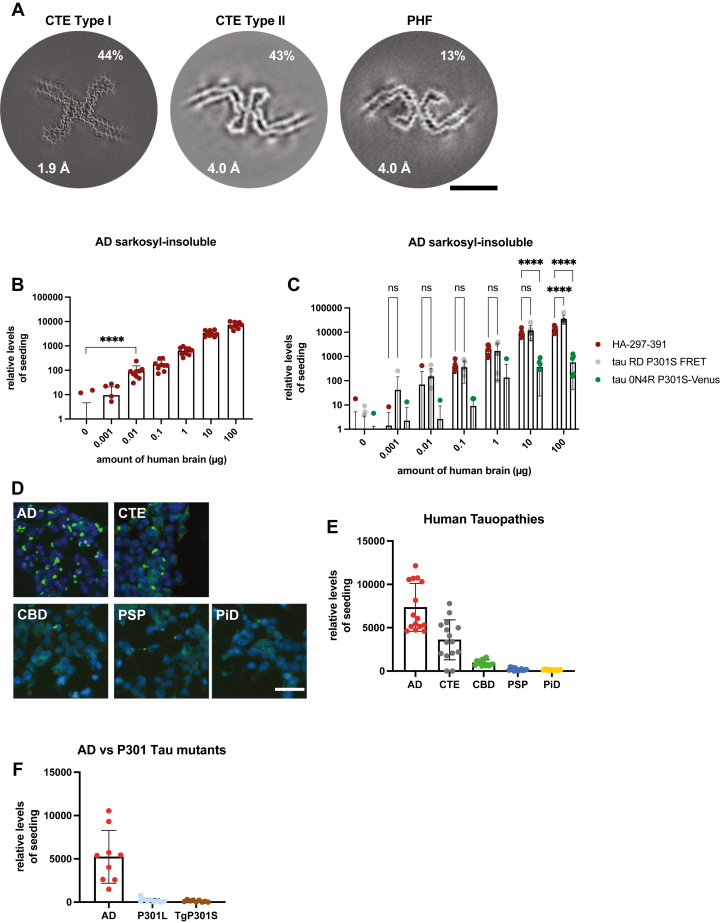
**HA-tau297–391 biosensor cells specifically detect seeds from 3R + 4R tauopathies.***A*, cross-sections perpendicular to the helical axis of the cryo-EM reconstructions of sarkosyl-insoluble filaments from the frontal cortex of a case of stage IV CTE, with a projected thickness of approximately one rung along the helical axis. Filament types are indicated, as are resolutions (in Å) and percentages of filament types. The scale bar represents 5 nm. *B*, seeding analysis of HA-tau297–391 biosensor cells challenged with increasing amounts of brain-derived sarkosyl-insoluble material from a case of AD. *C*, seeding efficiency with increasing amounts of sarkosyl-insoluble AD seeds of HA-tau297–391 cells and two previously reported biosensor cell lines. *D*, representative images of fixed HA-tau297–391 biosensor cells after seeding with sarkosyl-insoluble material extracted from AD, CTE, CBD, PSP, and PiD brains. Anti-HA antibody and Hoechst dye were used to label the overexpressed HA-tau297–391 and the cell nuclei, respectively. The scale bar represents 50 μm. *E* and *F*, quantification of HA-positive intracellular inclusions in HA-tau297–391 biosensor cells after seeding with sarkosyl-insoluble material extracted from human tauopathy brains and transgenic mice for human 0N4R tau P301S. The volumes of loaded samples were normalized based on the tau content of the final sarkosyl-insoluble fractions. See also Fig. S2. Image analysis included at least 6000 cells per condition from three experimental replicates. ^ns^P > 0.05 and ∗∗∗∗*p* < 0.0001 by one-way ANOVA with Tukey’s correction in *B* and *C*. Error bars denote standard deviations. AD, Alzheimer’s disease; CBD, corticobasal degeneration; CTE, chronic traumatic encephalopathy; HA, hemagglutinin; PiD, Pick’s disease; PSP, progressive supranuclear palsy; 3R, three repeat; 4R, four repeat.

We then assessed the seeding properties of the HA-tau297–391 biosensor cells using tau seeds extracted from human brains. Brain tissues from six individuals with different types of tauopathy (AD, CTE, Pick’s disease [PiD], progressive supranuclear palsy [PSP], corticobasal degeneration [CBD], and frontotemporal dementia and Parkinsonism linked to chromosome 17 with a P301L mutation in *MAPT*) were used for the extraction of sarkosyl-insoluble tau seeds of known structure. Frontal cortex from a case of AD (7) was used, as was frontal cortex from a case of PiD (29). Putamen from PSP-F case 1 (25), frontal cortex from CBD case 3 (30), and parietal cortex from P301L *MAPT* case 2 (31) were also used. In addition to these published cases, we used samples of frontal cortex from a previously reported 82-year-old male with a neuropathologically confirmed diagnosis of stage IV CTE (32). We analyzed the sarkosyl-insoluble fractions by cryo-EM and observed a mixture of type I and type II tau filaments with the CTE fold as well as a smaller number of Alzheimer's PHFs (Figs. 2*A* and S2, *A* and *B*). In addition to the human brain samples, we also extracted tau seeds from the brains of end-stage homozygous transgenic mice overexpressing full-length human 0N4R tau with the P301S mutation under the control of the murine *Thy1* promoter (33). These mice develop abundant filamentous inclusions of hyperphosphorylated tau and neurodegeneration, with an approximate lifespan of 7 to 8 months.

### Seeded aggregation in HA-tau297–391 biosensor cells

Initially, we challenged the cells with sarkosyl-insoluble species from a previously characterized case of AD (7) and fixed them 48 h later. HA-based immunofluorescence imaging indicated the dose-dependent formation of intracellular foci (Fig. S2*C*). Automated image analysis of the aggregated puncta identified a volume of 0.01 nl seeds per well (approximately 0.01 μg of AD brain tissue) as the detection threshold (Fig. 2*B*). Compared with previously established HEK293-based tau seeding reporter cell lines, our HA-tau297–391 biosensors outperformed 0N4R tau P301S-Venus cells (19) in detecting AD-derived seeds for all the titrated amounts, whereas the sensitivity was similar to that of the most widely used tau RD P301S FRET seeding biosensor system (18) (Fig. 2*C*).

We hypothesized that tau filaments extracted from the brains of individuals with CTE might also be able to induce seeding in our biosensor cell line, while this would not be the case for tau filaments with ordered cores comprising residues outside K297–E391. Apart from the CTE filaments that we analyzed by cryo-EM, we also used tau seeds from the brains of previously described individuals with 3R-only (PiD) or 4R-only (CBD and PSP) tauopathies (25, 29, 30). The amount of insoluble tau was normalized by dot blot analysis (Fig. S2*D*), and these seeds were used to challenge our biosensors. While the seeds from the 3R + 4R tauopathies induced the formation of intracellular puncta, seeds extracted from the brains of individuals with CBD, PSP, and PiD were much less effective (Fig. 2, *D* and *E*). CTE seeds induced the formation of intracellular HA-positive aggregates, but the level of seeding was approximately twofold lower than for AD seeds (Fig. 2*E*). A similar difference in seeding ability was observed when the cells were seeded with filaments that were assembled from recombinant tau297–391 under conditions that led to either AD PHFs or CTE filaments (Fig. S2*E*). We also used sarkosyl-extracted brain material from a human case with a P301L *MAPT* mutation and from mice transgenic for 0N4R P301S tau (31, 34). As expected from the presence of the proline 301 mutations and the observation that parts of the ordered cores of these filaments are located outside the sequence encoded by the construct that is overexpressed in the biosensor cells, both types of mutant tau seeds were over 50-fold less competent in seeding the assembly of HA-tau297–391 into filaments than tau seeds extracted from AD brains (Fig. 2*E*).

### Cryo-EM structure of seeded tau filaments in HA-tau297–391 biosensor cells

**Figure 3:**
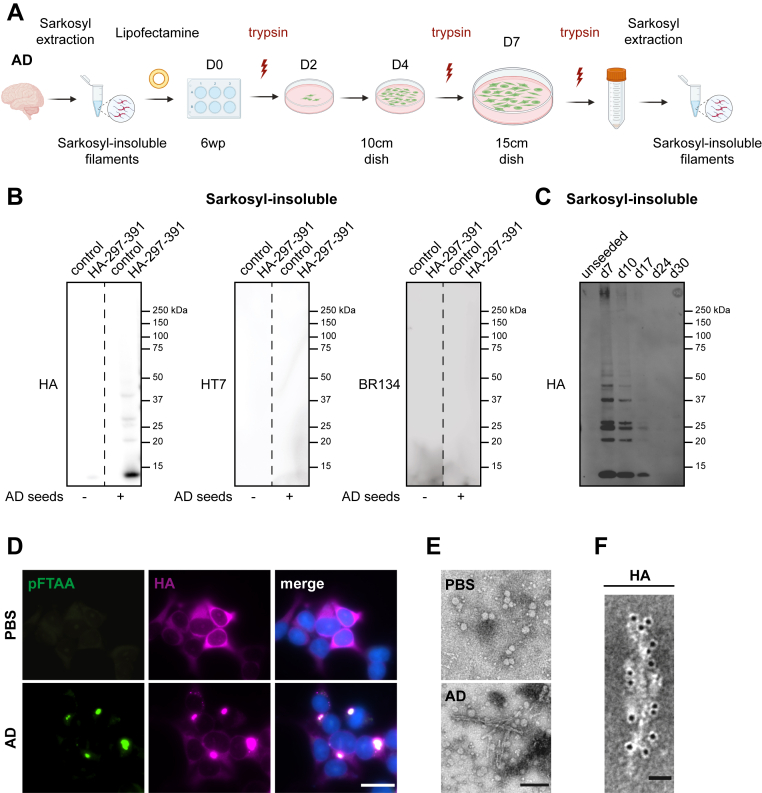
**Seeded assembly in HA-tau297–391 biosensor cells using Alzheimer's tau seeds.***A*, experimental protocol resulting in the sarkosyl-insoluble extraction of seeded samples. *B*, Western blot analysis of sarkosyl-insoluble extracts from control and HA-tau297–391 biosensor cells in the presence or absence of AD-tau seeds. Anti-HA antibody detects intracellularly expressed HA-tau297–391, whereas antitau antibodies HT7 and BR134 detect only the added seeds. *C*, Western blot analysis over time (days 7–30 after seeding) of sarkosyl-insoluble material extracted from HA-tau297–391 biosensors after seeding with AD seeds. *D*, representative immunofluorescence images from fixed HA-tau297–391 biosensor cells after challenge with PBS or AD-derived seeds. The cells were stained using the anti-HA antibody and the amyloid dye pFTAA, whereas Hoechst dye was used to label cell nuclei. The scale bar represents 25 μm. *E*, negative-stain TEM images of sarkosyl-insoluble extracts from HA-tau297–391 biosensor cells treated with either PBS or AD seeds. The scale bar represents 50 nm. *F*, immunogold labeling using the anti-HA antibody of a filament extracted from AD-seeded HA-tau297–391 biosensor cells. The scale bar represents 50 nm. AD, Alzheimer’s disease; HA, hemagglutinin; TEM, transmission electron microscopy.

For the structural analysis of assembled HA-tau297–391 species after seeding with AD-derived filaments, we maximized the yield of the cell-propagated aggregated species, while reducing the amount of exogenously added seeds. We allowed the cell population to expand for 7 days after seeding and performed three separate trypsinizations (Fig. 3*A*), since assembled tau is known to be sensitive to trypsin degradation (35). Immunoblots of sarkosyl-insoluble fractions showed a clear signal for HA-tagged material, but we were unable to identify the presence of full-length tau seeds using antibodies HT7 (epitope tau159–163) or BR134 (epitope tau428–441) (Fig. 3*B*). After the increase in intracellularly assembled HA-tau-297– 391 as a result of seeding with AD material, the cells showed a time-dependent decrease of insoluble HA-tagged tau species over time in culture (Fig. 3*C*), which was not because of fluctuations in total HA-tau297–391 levels (Fig. S3*A*). Moreover, the intracellularly formed HA-tagged tau assemblies stained positive for the pan-amyloid dye pFTAA (Fig. 3*D*) and induced robust second-generation seeding when reintroduced into the biosensors (Fig. S3*B*). Negative-stain transmission electron microscopy (TEM) showed the presence of filamentous assemblies that lacked a fuzzy coat and were decorated by gold-labeled HA antibody, unlike the tau seeds from AD brains (Fig. 3, *E* and *F*).

**Figure 4:**
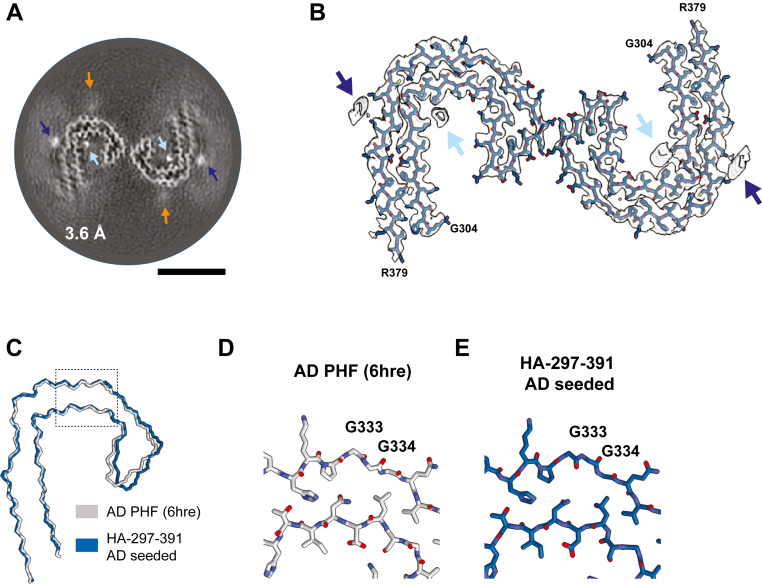
**Cryo-EM structure of filaments seeded by Alzheimer's tau filaments in HA-tau297–391 biosensor cells.***A*, cross-section perpendicular to the helical axis of the cryo-EM reconstructions of sarkosyl-insoluble filaments from AD-seeded HA-tau297–391 biosensor cells, with a projected thickness of approximately one rung along the helical axis. *Dark blue arrows* indicate additional densities in contact with K317 and K321, and *light blue arrows* indicate additional densities in proximity to H362 and K369. A weaker additional density indicated with the *orange arrow* is associated with residues H329 and K331. The scale bar represents 5 nm. *B*, cryo-EM density map of the sarkosyl-insoluble filaments extracted from AD-seeded HA-tau297–391 biosensor cells in *transparent gray*, superimposed by their refined atomic model. *C*, backbone ribbon of the atomic model of one protofilament from the doublet shown in *B* (*blue*), overlaid with the model of the PHF protofilament from AD brain (Protein Data Bank ID: 6HRE) (*gray*). *D* and *E*, detailed views of the ^332^PGGGQ^336^ motif with the same colors as in *C*. AD, Alzheimer’s disease; HA, hemagglutinin; PHF, paired helical filament.

We then used cryo-EM to determine the structure of HA-tau297–391 filaments extracted from the AD-seeded biosensor cells. Most isolated filaments consisted of two protofilaments with an approximate crossover distance of 900 Å (Fig. S4, *A* and *B*). Using helical reconstruction in RELION (36), we obtained a reconstruction at 3.6 Å resolution (Figs. 4*A* and S4*C*). The structures consisted of two identical C-shaped protofilaments that adopted a “head-to-head” orientation with C2 helical symmetry. The ordered core of each protofilament comprised residues G304–R379 that were arranged into eight β-strands and was almost identical (with an estimated all-atom RMSD of 1.3 Å) to the Alzheimer's tau fold of PHFs and SFs (Fig. 4, *B* and *C*). A similar head-to-head interaction between two protofilaments with the Alzheimer's fold has previously been described for an intermediate polymorph in the *in vitro* assembly of recombinant tau297–391 tau into PHFs (37). As in PHFs from human brains, the reconstruction of HA-tau297–391-seeded filaments showed additional densities in front of residues K317 and K321, as well as inside the C-shaped protofilament, close to residues H362 and K369 (Fig. 4, *A* and *B*, *dark and light blue arrows*, respectively). However, in contrast to PHFs from the human brain, reconstruction of the seeded aggregates also showed an additional density in front of residues H329 and K331 (Fig. 4*A*, *orange arrow*). Whereas the protofilament interface in PHFs is formed by the antiparallel stacking of residues ^332^PGGGQ^336^, with K331 orienting toward E338 on the opposite protofilament (7), the protofilaments in the “head-to-head” structure formed an electrostatic interaction between residues E342 and K343. It is therefore possible that the additional density in front of K331 prevented the formation of PHFs. In the absence of the second protofilament forming the PHF-related interface between residues ^332^PGGGQ^336^, the carbonyl group of G333 was facing inside the amyloid core (Fig. 4, *D* and *E*), whereas in PHFs from the human brain, the backbones of G333 and G334 form interprotofilament hydrogen bonds. A similar rearrangement of G333 has been reported for filaments from AD-seeded SH-SY5Y cells overexpressing HA-tagged full-length 1N3R tau (22).

### Short tau filaments from AD brains are the major seed-competent species

**Figure 5:**
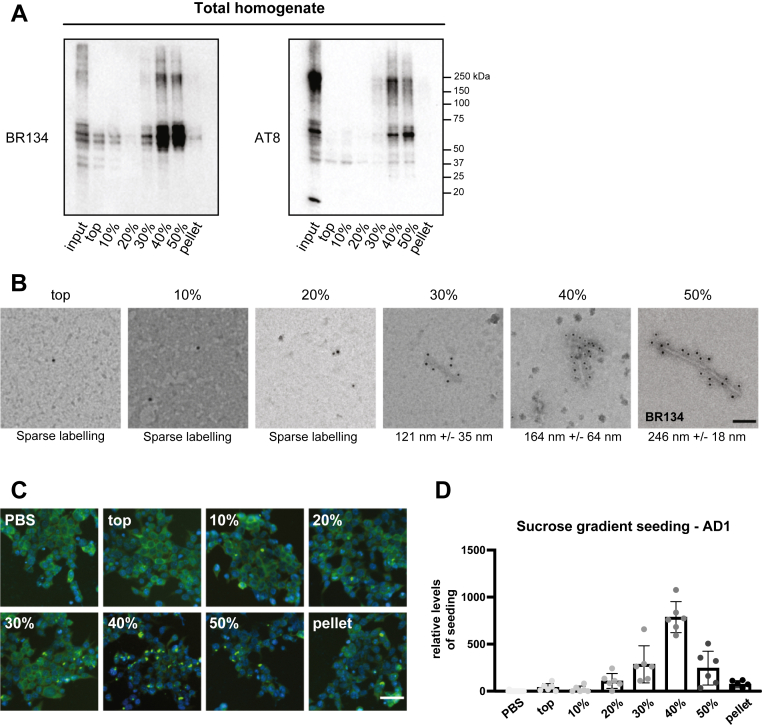
**Tau species in the 40% sucrose fraction from the frontal cortex of Alzheimer’s disease (AD) case 1 are the most seed competent.***A*, Western blot analysis (antitau antibodies BR134 and AT8) following SDS-PAGE of brain lysates from case AD1 fractionated by sucrose gradient centrifugation. *B*, immunoelectron microscopy of fractionated lysates with BR134. The measured filament lengths (30%, 40%, and 50%) are shown (n ≥ 7 filaments per fraction). The scale bar represents 100 nm. *C*, representative immunofluorescence images from fixed HA-tau297–391 biosensor cells challenged with PBS or the different sucrose gradient fractions. Anti-HA antibody and Hoechst dye were used for labeling of HA-tau297–391 and cell nuclei, respectively. The scale bar represents 50 μm. *D*, quantification of HA-positive intracellular inclusions in HA-tau297–391 biosensor cells following seeding with sucrose gradient fractionated AD1 brain lysates. The volume of the loaded sample for each condition was normalized based on the tau content of the final fraction. See also Fig. S5. Image analysis included at least 2000 cells per condition from three experimental replicates. Error bars denote standard deviations. AD, Alzheimer’s disease; HA, hemagglutinin.

Previous work on transgenic mouse models overexpressing human mutant P301S tau identified the filamentous species isolated from 40% sucrose gradient fractions as the most seed competent (15). Aiming to characterize the seed-competent tau species from AD brains, we fractionated brain lysates from case AD1 (7) by sucrose gradient centrifugation (38). Immunoblotting of total (BR134) and phosphorylated (AT8) tau identified most tau in the 40% and 50% fractions (Fig. 5*A*). By immunolabeling negative-stain TEM with BR134, the top, 10%, and 20% fractions contained only sparse labeling, whereas labeled filaments were detected in the 30%, 40%, and 50% fractions, with increasing filament lengths at higher densities (Fig. 5*B*). Following normalization by dot blot analysis (Fig. S5*A*), equal amounts of tau from each sucrose fraction were used as seeds on HA-tau297–391 biosensor cells that were fixed and stained (Fig. 5*C*). Automated image analysis identified the 40% sucrose fraction as the most seed competent, with an average filament length of 164 nm (Fig. 5*D*). The same series of experiments was performed using brain lysates from the frontal cortex of two additional previously described cases of AD (AD2 and AD3) (8). In line with the results from AD1, most tau species were in the 40% and 50% sucrose fractions (Fig. S5, *A* and *B*), with similar length ranges for isolated filaments (Fig. S5*C*). Moreover, seeding analysis of fractionated samples also identified the species isolated from the 40% sucrose fractions with a total average filament length of 175 nm as the most seed competent (Fig. S5*D*).

### Tau seeds from the AD brain are inactivated by sodium hypochlorite

**Figure 6:**
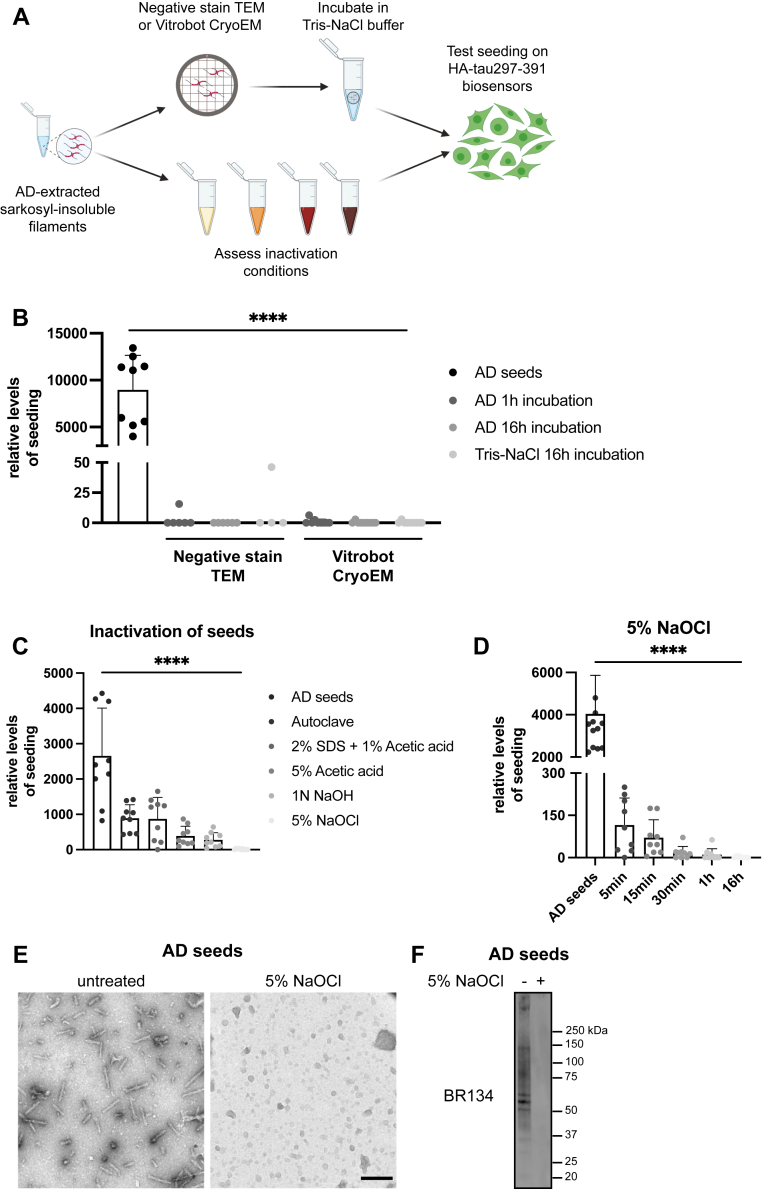
**Seeding ability of Alzheimer tau filaments on electron microscopy grids following inactivation treatments.***A*, schematic illustration of the experimental steps for testing the seeding properties of AD-extracted filaments following sarkosyl extraction. *B*, sarkosyl-insoluble seeds from AD were prepared for negative-stain TEM or cryo-EM, and the grids were incubated in a small volume of buffer to assess their seeding propensity in HA-tau297–391 cells. *C*, the AD-extracted seeds were incubated with various inactivation treatments for 1 h and then used for seeding experiments in the biosensor cells. Treatment with 5% NaOCl was the most effective in inactivating Alzheimer tau seeds. *D*, AD-derived seeds were inactivated with 5% NaOCl for different times. HA-positive intracellular inclusions in HA-tau297–391 biosensor cells were quantified for each condition. Image analysis for all graphs in this figure included at least 3900 cells per condition from three experimental replicates. *E*, negative-stain TEM images of AD-extracted seeds with and without treatment with 5% NaOCl for 1 h at room temperature. The scale bar represents 250 nm. *F*, AD-derived seeds were treated with 5% NaOCl for 1 h at room temperature and then immunoblotted with the antitau antibody BR134. ∗∗∗∗*p* < 0.0001 by one-way ANOVA with Tukey’s correction, and error bars denote standard deviations. AD, Alzheimer’s disease; HA, hemagglutinin; TEM, transmission electron microscopy.

The handling of material and instruments that have been in contact with assemblies of misfolded forms of the prion protein (PrP^Sc^) is tightly regulated to minimize transmission of the infectious proteinaceous agents. Although there is little evidence that filaments made of tau, α-synuclein, and amyloid-β do pose such a risk, regulatory frameworks have recently been proposed (39). In this context, we aimed to identify the potential risk of transmission in EM experiments and the optimal inactivation methods for tau seeds extracted from AD brains (Fig. 6*A*).

First, we assessed the seeding ability of tau filaments following deposition on EM grids, since tau assemblies have been shown to withstand formaldehyde fixation (40) and could therefore pose a risk during the handling of samples for light microscopy. Sarkosyl-insoluble tau filaments from a case of AD were blotted on negative stain and cryo-EM grids according to standard protocols (see the Experimental procedures section). Grids were then submerged in 10 μl of Tris–NaCl buffer and incubated at room temperature for 1 h or overnight. Using our biosensor cells, we did not detect seeding when using the buffer from the negative stain or the cryo-EM grids (Fig. 6*B*).

Despite the range of known inactivation methods for different prion strains (41, 42), inactivation protocols have so far been mostly tested for heparin-assembled recombinant tau filaments (43). However, since the amyloid structures of these filaments differ from those of tau filaments from human brains (44), the resulting conclusions may not be relevant for tau seeds from AD brains. We therefore screened various conditions by incubating AD-tau seeds from 1 mg of human brain tissue for 1 h at room temperature and then treated the biosensor cells at dilutions that did not induce a significant reduction in the number of cells (Fig. S6, *A* and *B*). Even though previous reports suggested autoclaving as an efficient inactivation method of AD-tau seeds (45), autoclaving or treatment with a combination of 2% SDS and 1% acetic acid was unable to fully eliminate the seeding ability of our samples. Harsh acidic or alkaline treatments were more effective, but the most effective inactivation method was the incubation with 5% sodium hypochlorite, which led to a 99.5% reduction in seeding ability (Fig. 6*C*). Inactivation of AD-derived tau seeds was titratable in terms of sodium hypochlorite concentrations and incubation times (Figs. 6*D* and S6*C*), with a 5 min incubation in 5% NaOCl, reducing the tau-seeding ability of the AD-derived material by 97% without affecting the viability of the cells (Fig. S6*D*). We also performed negative-stain TEM and Western blot analyses of the AD-tau seeds, but we were unable to detect even a trace of assembled tau after treatment with 5% NaOCl (Fig. 6, *E* and *F*). Therefore, we recommend the incubation of tau seeds with 5% NaOCl for 1 h as a means of inactivation. Sodium hypochlorite, also known as common household bleach, is a cost-effective and widely accessible chemical that oxidizes amino acids, which leads to protein fragmentation (46, 47).

## Discussion

Here, we describe an HEK293T-based seeding biosensor cell line that specifically detects seeds from 3R + 4R tauopathies and reproduces the Alzheimer's tau fold. The stably overexpressing HA-tagged tau fragment spans residues K297–E391 of wildtype 4R human tau, which was previously shown to assemble *in vitro* into PHFs identical with those found in the brains of individuals with AD (27, 48). We initially hypothesized that overexpression of HA-tau297–391 in cells might lead to *de novo* assembly into filaments but were unable to detect intracellular amyloid assemblies in the absence of added tau seeds. This difference may have resulted from lower cellular levels of tau297–391 expression compared with those needed for *in vitro* assembly and/or the activity of protein folding and degradation cellular machineries, preventing the assembly (49). However, upon exposure to recombinant PHFs or brain-derived AD-tau seeds, overexpressed HA-tau297–391 assembled into amyloid filaments. The detection sensitivity of our cells was similar to or superior to that of previously reported biosensors overexpressing 4R tau variants with the aggregation-prone mutation P301S (18, 19). Thus, our biosensor cell line ranks among the most sensitive available systems for the detection of AD-tau seeds.

Another property of our cell line is the consistent seeded assembly of tau amyloids based on sequence compatibility between the seed and the template. Thus, overexpressed HA-tau297–391 assembled almost exclusively upon seeding with tau seeds whose ordered core sequence matched the tau fragment, that is, from 3R + 4R tauopathies, such as AD and CTE. This property distinguishes our biosensor cell line from previously established cell models that overexpress tau fragments spanning residues 244 to 368 or 244 to 372 of 4R tau, which demonstrated limited specificity toward the isoform composition of tau seeds (18, 50). Apart from the type II P301T tau fold, all known tau amyloid structures from the human brain extend beyond the C terminus of these tau fragments (10, 25, 31); therefore, the structures of these tau seeds cannot be reproduced in these biosensor cells.

We also provide, for the first time, a structural characterization of the seeded aggregates in a tau seeding biosensor cell system. Upon seeding with AD-tau seeds, the major type of intracellular amyloid assemblies consisted of two identical protofilaments with the Alzheimer's fold. Previous reports from SH-SY5Y cells transiently overexpressing 1N3R tau that were treated with AD-tau seeds identified intracellular tau filaments consisting only of a single protofilament with the Alzheimer's fold (22). Potential factors contributing to these structural differences could be the cell type (HEK293T *versus* SH-SY5Y), the incubation period after seeding (7 days *versus* 3 days), and the amino acid sequence of the overexpressed template (tau297–391 tau *versus* full-length tau). The latter may be key because truncated tau has been shown to assemble more efficiently than full-length tau *in vitro* (51), whereas extension of the tau297–391 sequence toward the C terminus leads to the formation of single protofilaments with the Alzheimer's tau fold (27).

Even though the final packing of protofilaments did not match that observed in PHFs and SFs (7), the “head-to-head” structure reported here has previously been shown to be an intermediate in the *in vitro* assembly of tau297–391 into PHFs (37). It is unclear why the AD-seeded filaments did not form mature PHFs or SFs in the biosensor cells. One explanation might be that the period after seeding was not sufficient for completion of the assembly. In contrast to adult neurons, HEK293T cells are constantly dividing, which probably explains the observed reduction in the number of seeded filaments over multiple passages. Future studies will investigate the contribution of the cellular milieu to the seeded assembly of amyloid structures in nondividing neurons. Another explanation might be the presence of post-translational modifications or additional changes that are not found in the brains of AD patients. For example, in PHFs isolated from the brains of individuals with AD, residue K331 from one protofilament orients itself toward E338 from the second protofilament to further stabilize the final packing (7). However, in the AD-seeded HA-tau297–391 filaments, there was an additional weaker density in front of H329 and K331, which may have obstructed the formation of mature PHFs.

In conclusion, we describe a HEK293T-based tau seeding biosensor cell line that specifically responds to seeds composed of 3R + 4R tau filaments, with the seeded filaments reproducing the Alzheimer's tau fold. Apart from AD and CTE, 3R + 4R tau filaments are found in several other tauopathies, including primary age–related tauopathy (52), familial British and Danish dementias (25), some prion protein amyloidoses (53), frontotemporal dementias caused by missense mutations V337M and R406W in *MAPT* (54), subacute sclerosing panencephalitis, amyotrophic lateral sclerosis/Parkinsonism–dementia complex of Guam and the Kii peninsula (55), and in vacuolar tauopathy (56). This improved biosensor cell line can be used for identifying and quantifying the levels of 3R + 4R tau seeds in these diseases as well as for a better understanding of the mechanisms underlying seeded tau aggregation.

## Experimental procedures

### Cell culture media and chemicals

Cell culture media were purchased from Thermo Fisher Scientific and chemicals were purchased from Merck, unless stated otherwise.

### Cell lines

HEK293T (CRL-3216) and tau RD P301S FRET (18) (CRL-3275) cells were purchased from American Type Culture Collection, and the 0N4R tau P301S-Venus cells were provided by Dr W.A. McEwan (19). All cell lines were maintained in complete medium (Dulbecco's modified Eagle's medium high glucose GlutaMAX pyruvate, supplemented with 10% fetal bovine serum [FBS], 100 U/ml penicillin, and 100 μg/ml streptomycin) and grown at 37 °C and 95% O_2_/5% CO_2_.

### Lentivirus-mediated generation of HA-tau297–391 biosensor cells

HEK293T cells stably expressing HA-tau297–391 were generated with lentiviral-mediated transduction according to previously established protocols (57). The cDNA construct was amplified using the primers 4R-HA_K297_EcoRI_fw: CTGACTGACTGAGAATTCGCCACCATGTACCCATACGATGTTCCAGATTACGCTATCAAACACGTCCCGGGAGGC and 4R-E391_XhoI_rev: CTGACTGACTGACTCGAGTCTACTACTCCGCCCCGTGGTCTGTC. The resulting PCR products were cloned into the lentiviral transfer plasmid pHR-SFFV_3C-Twin-Strep (Addgene plasmid #113900), using the indicated restriction enzymes (New England Biolabs) to replace the 3C-Twin-Strep sequence. Lentiviral particles were produced using the second-generation envelope plasmid pMD2.G (Addgene plasmid #12259) and the second-generation packaging plasmid psPAX2 (Addgene plasmid #12260). Control HEK293T cells were plated in a 10 cm dish at a density of 300,000 cells/ml, and the next day, their medium was replaced with transduction medium (Dulbecco's modified Eagle's medium high glucose GlutaMAX pyruvate supplemented with 2% FBS, 100 U/ml penicillin, and 100 μg/ml streptomycin). For plasmid transfection, 10 μg of each plasmid in a 1:1:1 ratio was diluted in Opti-MEM Reduced Serum Medium and cotransfected in the cells using Lipofectamine 3000 (Thermo Fisher Scientific) according to the manufacturer’s instructions. After 72 h, the supernatants were clarified using a 0.45 μm filter, mixed with Polybrene (Merck; TR-1003-G) at a final concentration of 10 μg/ml and used to transduce the control HEK293T cells. The cells were expanded, and the transduction procedure was repeated twice before using the cells. Expression and transduction efficiencies were assessed by Western blot and immunofluorescence analyses.

### Expression and purification of wildtype and P301S tau297–391

The expression of recombinant tau297–391 and tau297–391 P301S was carried out in *Escherichia coli* BL21 (DE3)-gold cells (Agilent Technologies; catalog no.: 200131), as described previously (27). Briefly, one agar plate of cells was resuspended in 1 l of 2xTY (tryptone yeast) supplemented with 100 mg/l ampicillin and grown to an absorbance of 0.8 at 600 nm. Cells were induced by the addition of 1 mM IPTG for 4 h at 37 °C, collected by centrifugation (4000*g* for 20 min at 4 °C), resuspended in 10 ml/g pellet of washing buffer (50 mM Mes at pH 6.0; 10 mM EDTA; 10 mM DTT), supplemented with 0.1 mM phenylmethylsulfonyl fluoride and cOmplete EDTA-free protease cocktail inhibitors, and heated at 95 °C for 5 min. Cell lysis was carried out using sonication (at 40% amplitude using a Sonics VCX-750 Vibracell Ultra Sonic Processor for 7 min, 5 s on/10 s off). Lysed cells were centrifuged at 20,000*g* for 35 min at 4 °C, filtered through 0.45 μm cutoff filters, and loaded onto a HiTrap CaptoS 5-ml column (Cytiva; catalog no.: 11001303) for cation exchange. The column was washed with 10 volumes of washing buffer and eluted using a gradient of washing buffer containing 0 to 1 M NaCl. Fractions of 3.5 ml were collected and analyzed by SDS-PAGE followed by staining with Instant Blue Coomassie (Expedeon; catalog no.: 194-ISB1L). Protein-containing fractions were pooled and precipitated using 0.38 g/ml ammonium sulfate and left on a rocker for 30 min at 4 °C. Precipitated proteins were then centrifuged at 20,000*g* for 35 min at 4 °C, resuspended in 2 ml of 10 mM phosphate buffer at pH 7.2 with 10 mM DTT, and loaded onto a HiLoad 16/600 Superdex 75 pg size-exclusion column (Cytiva; 28989333). Size-exclusion fractions were also analyzed by SDS-PAGE, and protein-containing fractions were pooled and concentrated to 20 mg/ml using molecular weight concentrators with a cutoff filter of 3 kDa. Purified protein samples were flash frozen in 50 to 100 μl aliquots for future use. Protein concentrations were determined using a NanoDrop2000 (Thermo Fisher Scientific).

### Assembly of recombinant tau filaments

Assembly reactions were carried out in aliquots of 40 μl of purified monomeric tau297–391 and tau297–391 P301S at 6 mg/ml in a 384-well microplate that was sealed and placed in a Fluostar Omega (BMG Labtech). PHF reactions were carried out in 10 mM phosphate buffer at pH 7.2, 100 mM MgCl_2_, and 10 mM DTT, whereas CTE reactions were carried out in 50 mM phosphate buffer at pH 7.2, 150 mM NaCl, and 10 mM DTT. The assembly reaction of tau297–391 P301S was carried out in 50 mM phosphate buffer at pH 7.2, 150 mM NaCl, and 10 mM DTT. Reactions were carried out for 48 h using 200 rpm orbital shaking at 37 °C.

### Case of CTE

The proband played American football between the ages of 13 to 25 years, including 3 years in the National Football League. He suffered around 100 concussions during this time, some with loss of consciousness. He developed progressive cognitive impairment and was diagnosed with dementia at age 67. Around this time, personality changes also became manifest. In subsequent years, the proband experienced frequent falls, developed a tremor, and a shuffling gait. A diagnosis of Parkinson’s disease was made at age 78. He died aged 82 of complications from Parkinson’s disease. At autopsy, there was a severe panlobar atrophy of the cerebral cortex, together with marked ventricular enlargement. Multiple CTE lesions and abundant neurofibrillary tangles were in evidence. Hippocampus, entorhinal cortex, and amygdala were also severely atrophic, accompanied by abundant neurofibrillary pathology. Rounded TDP-43-immunoreactive neurites were present in the hippocampus and amygdala. There was severe neuronal cell loss in the substantia nigra, accompanied by abundant neurofibrillary tangles and rare Lewy pathology. These neuropathological changes conform to stage IV CTE (32).

### Transgenic mice

Animal experiments were carried out in accordance with the UK Animals (Scientific Procedures) Act of 1986, with local ethical approval (MRC Laboratory of Molecular Biology Animal Welfare and Ethical Review Body). Tg2541 mice express full-length human tau (0N4R) with the P301S mutation under the control of the murine Thy1 promoter on a mixed C57BL/6 × CBA background (33). Brainstem and spinal cord from symptomatic mice (25 weeks of age) were used to extract sarkosyl-insoluble tau seeds.

### Extraction of tau filaments

Tissues were homogenized using a Polytron (PT 2500 E; Kinematica AG) in 15 v/w buffer A (10 mM Tris–HCl [pH 7.4], 800 mM NaCl, 10% sucrose, and 1 mM EGTA, 0.1% sarkosyl) supplemented with cOmplete EDTA-free Protease Inhibitor Cocktail and PhosSTOP Phosphatase Inhibitor Cocktail, brought to 1% sarkosyl, and incubated for 30 min at 37 °C. The homogenates were centrifuged at 10,000*g* for 10 min at 4 °C, and the supernatants were passed through a 70 μm cell strainer before ultracentrifugation at 150,000*g* for 1 h at 4 °C. The pellets were resuspended in 250 μl buffer A containing 1% sarkosyl and diluted threefold in buffer B (50 mM Tris–HCl [pH 7.4], 150 mM NaCl, 10% sucrose, and 0.2% sarkosyl). The samples were then sonicated with a Microson XI-2000 Ultrasonic Cell Disruptor (Misonix) for 30 s, and all samples were spun again at 150,000*g* for 1 h at 4 °C. The final pellets were resuspended in 50 μl/g of Tris–NaCl buffer (20 mM Tris–HCl [pH 7.4] and 100 mM NaCl).

Seeded cells were harvested by trypsinisation, and the cell pellets were vigorously resuspended in 15 volumes of buffer A already containing 1% sarkosyl. The lysates were sonicated with a Microson XI-2000 Ultrasonic Cell Disruptor (Misonix) for 15 s to fully disrupt the cells and then incubated for 30 min at 37 °C. The samples were centrifuged at 10,000*g* for 10 min at 4 °C, followed by ultracentrifugation at 150,000*g* for 1 h at 4 °C. The pellets were resuspended in 250 μl buffer A containing 1% sarkosyl and diluted threefold in buffer B (50 mM Tris–HCl [pH 7.4], 150 mM NaCl, 10% sucrose, and 0.2% sarkosyl). They were then centrifuged again at 150,000*g* for 1 h at 4 °C, and the final pellets were resuspended in 50 μl/g of Tris–NaCl buffer (20 mM Tris–HCl [pH 7.4] and 100 mM NaCl). For Western blot analysis, 4.5 μl of the resuspended sarkosyl-insoluble pellets were diluted 10-fold in Tris–NaCl buffer, whereas 45 μl from the total lysates and soluble fractions were kept. In both cases, 15 μl of 4× NuPAGE LDS sample buffer (Thermo Fisher Scientific; NP0007) containing 2 mM β-mercaptoethanol were added, and the final samples were boiled at 100 °C for 5 min before subjecting 10 μl to SDS-PAGE.

### Seeding assays

Seeding experiments were performed as described (19), with small modifications. Approximately 15,000 cells/well were plated in black 96-well plates that had been precoated with poly-d-lysine (Merck; A-003-E, final coating concentration of 50 mg/ml) and left to adhere for 20 h. The next day, the cells were rinsed with PBS, supplemented with 50 μl/well Opti-MEM Reduced Serum Medium, and placed back in the incubator during the preparation of tau seed–Lipofectamine mixtures. Tau seeds were diluted to the indicated amounts and mixed with 1 μl of Lipofectamine 2000 (Thermo Fisher Scientific; catalog no.: 11668019) in a final volume of 50 μl Opti-MEM Reduced Serum Medium for each well. The mixtures were incubated for 10 min at room temperature and then added to the cells. At least three equivalent PBS-mock controls were included as the untreated conditions. Following an incubation of 1 h, 100 μl/well of cOmplete medium were added to stop the transfection process. The cells were cultured for 48 h after the addition of tau seeds and were then fixed with cold methanol for 3 min at room temperature. Immunofluorescence labeling of the overexpressed HA-tau297–391 was done *via* incubation with a primary anti-HA (HA.11; BioLegend, 16B12) antibody followed by a secondary Alexa647 goat–anti-mouse IgG antibody (Thermo Fisher Scientific; catalog no.: A-21235), both diluted in IF blocking buffer (1× PBS, 5% FBS) for 1 h at room temperature. Nuclei were stained with 1 μg/ml Hoechst dye 33342 (Thermo Fisher Scientific; catalog no.: H3570) diluted in 1× PBS for 10 min, and images were acquired at 405 and 647 nm on a Ti2-E High Content Microscope (Nikon). Six or nine fields of view per well were read in a horizontal serpentine acquisition mode with a 10× objective, and the downstream analysis was performed using the Fiji software (28).

### Image analysis

Image analysis was performed in the open-source Fiji software (28), and scripts used for this work are available on GitHub (https://github.com/tkatsine/Nuclear-counting.git and https://github.com/tkatsine/Counting-protein-aggregates.git). For nuclear counting, images acquired at 405 nm were locally subtracted for background using the Rolling ball algorithm, and cells were segmented based on nuclear staining using the Median Filter and Find Maxima tools, with the option of “Segmented particles above lower threshold” option activated. Seeded aggregates in the 647 nm images were detected and quantified using the ComDet plugin (58) in Fiji, by using the parameters that allowed the detection of the minimum possible positive puncta in the unseeded control. Relative levels of seeding were calculated as the number of detected puncta in each field of view, normalized to the corresponding number of cells, and then compared with the untreated control.

### Seeding of HA-tau297–391 biosensor cells for cryo-EM analysis

Approximately 600,000 cells were plated in 6-well plates and left to adhere for 20 h. For each well, 5 μl of AD-tau seeds (approximately 500 μg of AD brain tissue) were mixed and incubated with 15 μl of Lipofectamine 2000 (catalog no.: 11668019) in 1 ml of Opti-MEM Reduced Serum Medium for 10 min at room temperature. An equivalent PBS-mock control was included as the untreated condition. At the end of the 10-min incubation, the medium was removed from the cells, and AD-tau seeds or PBS-mock control were added for 1 h. Then, 1 ml of the cOmplete medium was added to each well to stop the transfection. Cells were cultured for 48 h, and each well was treated with 0.5 ml of 0.25% trypsin–EDTA solution for 3 min at 37 °C, followed by inactivation with 2.5 ml of cOmplete medium. The detached cells were transferred to 10 cm dishes and allowed to expand for 48 h, after which they were trypsinized again and transferred to 15 cm dishes for another stage of expansion for a further 72 h. At the end of the cell expansion (7 days postseeding), cells were harvested using the final stage of trypsinization, and the weights of the final cell pellets were recorded. Typically, cells from two wells of a 6-well plate led to five 15 cm dishes that altogether yielded approximately 0.5 g of final cell mass. For the analysis of AD-seeded HA-tau297–391 over time, cells were seeded in 6-well plates and expanded in 10 cm dishes as described above. Cells were maintained and passaged in that format, while being harvested at the indicated time points for sarkosyl-insoluble extraction.

### Wide-field immunofluorescence microscopy

Approximately 40,000 control and HA-tau297–391 biosensor cells were plated in glass-bottom 8-well chambers (iBidi; catalog no.: 80827), precoated with poly-d-lysine (catalog no.: A-003-E, final coating concentration of 50 mg/ml). The cells were fixed after 48 h with cold methanol for 3 min at room temperature and stained against HA (Merck; catalog no.: 11867423001) and tubulin (DHSB; catalog no.: 12G10), followed by goat–anti-rat Alexa488 (Thermo Fisher Scientific; catalog no.: A-11006) and goat–anti-mouse Alexa647 (Thermo Fisher Scientific; catalog no.: A-21235) secondary antibodies. Both primary and secondary antibodies were diluted in IF blocking buffer and incubated for 1 h at room temperature. Nuclei were stained with 1 μg/ml Hoechst dye 33342 (H3570) for 10 min at room temperature. Images were acquired at 405, 488, and 647 nm on a Ti2-inverted fluorescence microscope (Nikon) using a 10× objective.

For pFTAA imaging, HA-tau297–391 biosensor cells were plated in 6-well plates, treated with AD-tau seeds or PBS, and expanded into 10 cm dishes as described above. During the final expansion step in 15 cm dishes, approximately 30,000 cells were plated in glass-bottom 8-well chambers (iBidi; catalog no.: 80827) precoated with poly-d-lysine (A-003-E, final coating concentration of 50 mg/ml). Cells were cultured for another 72 h (7 days post seeding) and then fixed with methanol for 3 min at room temperature. Immunofluorescence labeling of the overexpressed HA-tau297–391 was done *via* incubation with a primary anti-HA antibody (HA.11; BioLegend, 16B12) followed by a secondary Alexa647 anti-mouse IgG antibody (Thermo Fisher Scientific; A-21235), both diluted in blocking buffer (1× PBS, 5% FBS) for 1 h at room temperature. Staining with pFTAA (59) was performed by incubating the fixed cells in a final concentration of 33 nM pFTAA diluted in PBS for 30 min at room temperature. Nuclei were stained with 1 μg/ml Hoechst dye 33342 (H3570) for 10 min at room temperature. Images were acquired at 405, 488, and 647 nm on a Ti2-inverted fluorescence microscope (Nikon) using a 63× objective.

### SDS-PAGE and Western blotting

Analysis of HA-tau297–391 expression was carried out from total cell lysates. Approximately 2 × 10^6^ control or HA-tau297–391 biosensor cells were lysed on ice for 15 min in 500 μl 1× radioimmunoprecipitation buffer (Merck; R0278) supplemented with cOmplete EDTA-free Protease Inhibitor Cocktail (Merck; 11873580001) and PhosSTOP Phosphatase Inhibitor Cocktail (Merck; 4906845001). Lysates were centrifuged (14,000*g*, 15 min, at 4 °C), and 450 μl of clarified lysate was mixed with 150 μl 4× NuPAGE LDS sample buffer (Thermo Fisher Scientific; NP0007) containing 2 mM β-mercaptoethanol. The final samples were boiled at 100 °C for 5 min, and 12 μl were analyzed by SDS-PAGE.

SDS-PAGE for samples containing HA-tau297–391 was performed using NuPAGE Bis–Tris 4% to 12% gels (Thermo Fisher Scientific; NP0324BOX) in MES–SDS running buffer for 40 min at 200 V, whereas the sucrose gradient samples were analyzed in NuPAGE Bis–Tris 4% to 12% gels in Mops–SDS running buffer for 55 min at 200 V. Gels were electroblotted onto a 0.2 mm nitrocellulose membrane (Bio-Rad; catalog no.: 1704158) using the Bio-Rad Transblot Turbo Transfer System. The transferred membranes were blocked in 3% bovine serum albumin (Fisher BioReagents; BP9702) diluted in PBS with 0.2% Tween-20 (PBST) for 1 h at room temperature and then incubated with the primary antibodies overnight at 4 °C. The following primary antibodies were used for Western blot analysis: anti-HA (1:3000 dilution, HA.11; BioLegend, 16B12), the phosphorylation-independent antibodies against tau HT7 (1:3000 dilution; Thermo Fisher Scientific, MN1000), BR135 (1:3000 dilution, residues 323–335), and BR134 (1:3000 dilution, residues 428–441) (5), the phosphorylation-dependent antibody against tau AT8 (1:1500 dilution; Thermo Fisher Scientific, MN1020) as well as the loading control GAPDH (1:3000 dilution; Thermo Fisher Scientific, MA5-15738). After repeated washes with PBST, the membranes were incubated with Alexa-conjugated secondary antibodies (Thermo Fisher Scientific; A-11008, A-21244, A-21235, and A-21121) at a 1:2000 dilution for 1 h at room temperature. Finally, membranes were imaged using a ChemiDoc gel imager equipment (Bio-Rad).

### Sucrose gradient centrifugation

Frontal cortex from three previously described cases of AD (7, 8) was homogenized using a Polytron (PT 2500 E; Kinematica AG) in sterile homogenization buffer (50 mM Tris–HCl [pH 7.4], 100 mM NaCl) supplemented with 1× cOmplete EDTA-free Protease Inhibitor Cocktail (nine volumes/g tissue). Homogenates were centrifuged at 13,000*g* for 10 min at 4 °C. Supernatants were collected and stored in aliquots at −80 °C until use.

For sucrose gradient fractionation, 10%, 20%, 30%, 40%, and 50% sucrose fractions were prepared in homogenization buffer and layered in 4 ml centrifuge tubes (Beckman Coulter; #344062). The volumes were 0.4 ml 50%, 0.4 ml 40%, 0.4 ml 30%, 0.4 ml 20%, and 1 ml 10% sucrose, layered in decreasing density. Brain lysates from the sporadic AD cases (15 mg in 1 ml) were added on top of the gradient, and the tubes were centrifuged at 250,000*g* in an SW 60 Ti rotor for 4 h at 20 °C. The fraction remaining at the top of the sucrose gradient following centrifugation was called “Top.” The pellet that formed at the bottom of the tube was dislodged and disrupted by probe sonication into 0.5 ml PBS. All fractions were collected and stored at −80 °C.

### Negative-stain EM

Assembled recombinant tau filaments, sarkosyl-insoluble species from seeded cells, and sucrose gradient fractions from AD cases were applied to glow-discharged (Edwards S150B; Edwards High Vacuum International) 400 mesh formvar/carbon film–coated copper grids (EM Sciences; CF400-Cu) for 45 s. For negative-stain analysis, the excess liquid was removed, the grids were stained with 2% uranyl acetate for 45 s, and air-dried for 30 min before image acquisition.

For immunogold labeling, grids with deposited samples were blocked at room temperature for 10 min with TEM Blocking buffer (PBS + 0.1% cold fish skin gelatin [G7041; Merck]), followed by incubation with the indicated primary antibodies (1:50 dilution) diluted in TEM Blocking buffer for 1 h at room temperature. The grids were subsequently washed with blocking buffer and incubated with 10 nm gold-conjugated anti-mouse (Merck; G7652) or anti-rabbit (Merck; G7402) IgG secondary antibody for 1 h at room temperature and diluted 1:20 in TEM Blocking buffer. The grids were finally washed with water, stained with 2% uranyl acetate for 45 s, and air-dried for at least 30 min before imaging. Images were acquired at 4400× and 6500× with a defocus value of −1.4 mm with a Gatan Orius SC200B detector using a Tecnai G2 Spirit at 120 kV.

### Tau filament lengths after sucrose gradient fractionation

The samples were prepared, labeled, and the images acquired as described above. The decorated filaments were manually selected, and their lengths were measured using the Fiji software. For AD1, nine filaments were measured in the 30% sucrose fraction (average length, 120.7 nm; SD, 34.7 nm), 13 filaments in the 40% sucrose fraction (average length, 164.3 nm; SD, 64.3 nm), and 7 filaments in the 50% sucrose fraction (average length, 245.8 nm; SD, 124.2 nm). For AD2, 20 filaments were measured in the 30% sucrose fraction (average length, 93 nm; SD, 27.1 nm), 25 filaments in the 40% sucrose fraction (average length, 197.2 nm; SD, 67 nm), and 24 filaments in the 50% sucrose fraction (average length, 249.5 nm; SD, 143.3 nm). For AD3, 7 filaments were measured in the 30% sucrose fraction (average length, 107.4 nm; SD, 42.1 nm), 8 filaments in the 40% sucrose fraction (average length, 166.8 nm; SD, 128.6 nm), and 17 filaments in the 50% sucrose fraction (average length, 196.3 nm; SD, 61.7 nm). The top, 10%, 20%, and pellet fractions contained only sparse labeling.

### Dot blot analysis

Sarkosyl-insoluble samples from tauopathy cases were diluted 2500- and 5000-fold, whereas sucrose gradient fractions from the three AD cases were diluted 500-fold. The samples were applied in triplicate to a 0.2 mm nitrocellulose membrane (Bio-Rad; catalog no.: 1620112) using the Bio-Dot microfiltration apparatus (Bio-Rad; catalog no.: 1703938) according to the manufacturer’s instructions. The membranes were blocked in 3% bovine serum albumin (Fisher BioReagents; BP9702) diluted in PBST for 1 h at room temperature and then incubated with the phosphorylation-independent primary antibodies BR134 (1:3000 dilution) or HT7 (1:2000 dilution; Thermo Fisher Scientific, MN1000) overnight at 4 °C. The next day, membranes were washed three times with PBST and incubated for 1 h at room temperature with secondary Alexa-conjugated secondary antibodies (Thermo Fisher Scientific; A-11008, A-21244, A-21235, and A-21121) at a 1:2000 dilution for 1 h at room temperature. Finally, the membranes were imaged using a ChemiDoc gel imager system (Bio-Rad), and the dots were quantified using the Fiji software (28).

### Cryo-EM

Sarkosyl-insoluble fractions were centrifuged at 3000*g* for 3 min at room temperature. One microliter of the supernatant was diluted threefold, and a volume of 3 μl was applied to glow-discharged (Edwards S150B; Edwards High Vacuum International) holey carbon grids (Quantifoil Au R1.2/1.3, 300 mesh; Quantifoil) that were plunge-frozen in liquid ethane using a Vitrobot Mark IV (Thermo Fisher Scientific) at 100% humidity and 4 °C. Cryo-EM images were acquired using EPU software on a Titan Krios microscope (Thermo Fisher Scientific) operated at 300 kV and equipped with a Falcon-4i detector and a total dose of 40 electrons per Å^2^ at a pixel size of 0.47 Å with a quantum energy filter with a slit width of 10 eV or a Gatan K3 detector and a total dose of 30 electrons per Å^2^ at a pixel size of 0.93 Å with a quantum energy filter with a slit width of 20 eV. See also Table S1 for additional cryo-EM data acquisition parameters.

### Helical reconstruction

Datasets were processed in RELION using standard amyloid helical reconstruction procedures (36). Movie frames were gain-corrected, aligned, and dose-weighted using RELION’s own motion correction program (60). Contrast transfer function was estimated using CTFFIND-4.1 (61). Filaments from the AD-tau–seeded HA-tau297–391 biosensor cells were picked manually, whereas filaments from the CTE cases were automatically picked using a modified version of Topaz (62). Particles were extracted in box sizes of 768 or 1024 pixels and downscaled to 256 pixels. Reference-free 2D classification was used to distinguish different filament types, discard suboptimal images, and measure cross-over distances for initial model calculation using relion_helix_inimodel2d (63). Selected class averages were re-extracted with a box size of 400 pixels. Three-dimensional autorefinements were performed with optimization of the helical twist and rise parameters. Bayesian polishing was used to improve the resolution (64). The final maps were sharpened using postprocessing, and resolution estimates were calculated on the basis of the Fourier shell correlation between two independently refined half-maps at 0.143 (Fig. S4) (65).

### Model building

Atomic models of tau filaments from AD-seeded HA-tau297–391 biosensor cells were built using the initial model with Protein Data Bank accession code 7QKI. Atomic refinement was performed in ISOLDE using three rungs. Dihedral angles from the middle rung were set as a template for the rungs above and below. For each structure, Phenix refinement was performed on the first half-map. The resulting model was compared with this half-map and the second half-map to confirm the absence of overfitting (Fig. S4).

### Assessing seeding ability after deposition on EM grids

Sarkosyl-insoluble fractions were prepared from the frontal cortex of a case of AD. The samples and buffer only were diluted fivefold, and then 3 μl were applied to glow-discharged TEM and cryo-grids. Negative staining and plunge freezing in liquid ethane were performed as described above. Grids were then submerged in 10 μl of Tris–NaCl buffer for 1 h or 16 h at room temperature, the supernatant was collected and used as seeding material to treat the HA-tau297–391 biosensor cells. An equivalent amount of the starting material (AD seeds) was used as a positive control.

### Inactivation of tau seeds extracted from the AD brain

Tau seeds from 1 mg of frontal cortex from a case of AD were subjected to an autoclaving cycle of 50 min at 121 °C and 15 psi or incubated with various inactivation chemicals at the indicated concentrations at room temperature for 1 h, unless stated otherwise. The samples were then diluted 100-fold in Tris–NaCl buffer and used for seeding experiments in HA-tau297–391 cells. An equivalent amount of untreated tau seeds corresponding to 10 μg of human brain tissue was used as a positive control.

## Data availability

All data are contained within this article. The cryo-EM map and the structural model of the head-to-head tau filaments with the Alzheimer’s fold from the AD-seeded HA-tau297–391 biosensor cells are deposited into the Electron Microscopy Databank (EMD-54909) and the Protein Data Bank (9SHS).

## Supporting information

The article contains supporting information.

## Conflict of interest

The authors declare that they have no conflicts of interest with the contents of this article.
