# Supplementary material for: Seeding biosensor cell line that reproduces the Alzheimer tau fold
Source: J Biol Chem. 2025 Nov 17;301(12):110952. doi: 10.1016/j.jbc.2025.110952 (PMC12765094; doi:10.1016/j.jbc.2025.110952)
Supplement: Supporting information [file mmc1.pdf]

Supplementary figure 1

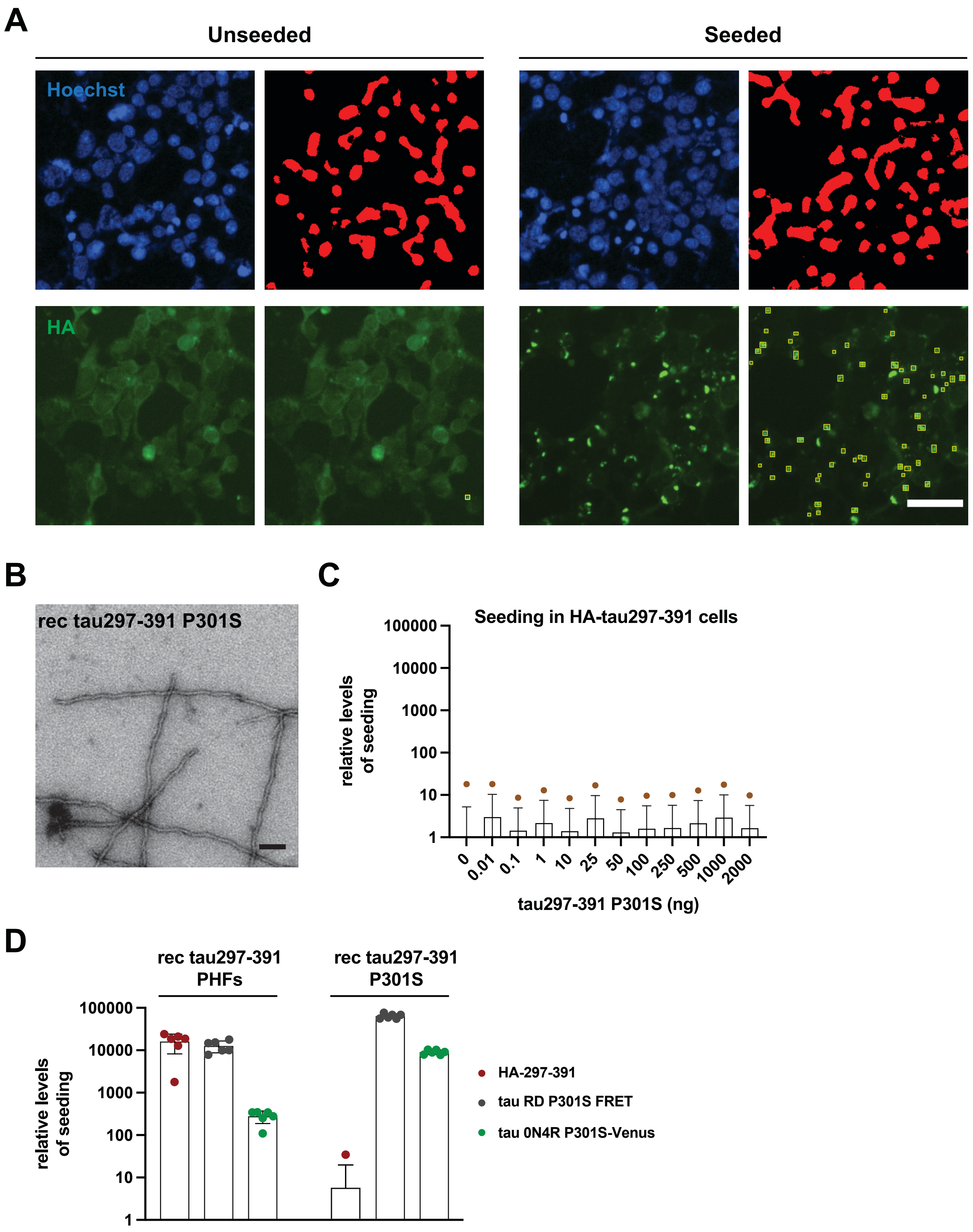

Figure S1. Image analysis segmentation and seeding propensity of recombinant tau297-391 P301S filaments

(A) Representative immunofluorescence images before and after segmentation and particle selection of unseeded and seeded HA-tau297-391 cells. The biosensor cells were labelled with an anti-HA antibody and Hoechst dye for staining the HA-tau297-391 protein and the cell nuclei, respectively. Scale bar, 50 µm. (B) Representative image of recombinant tau297-391 P301S filaments by TEM. Scale bar, 100 nm. (C) Quantification of HA-positive intracellular inclusions in HA-tau297-391 biosensor cells after seeding with increasing amounts of recombinant tau297-391 P301S filaments. Image analysis included at least 15,000 cells per condition from 3 experimental replicates. (D) Seeding abilities of 250 ng recombinant tau297-391 PHFs and tau297-391 P301S filaments were compared in three different tau seeding biosensor cell lines. Image analysis included at least 5,500 cells per condition from three experimental replicates. Error bars denote standard deviations.

Supplementary figure 2

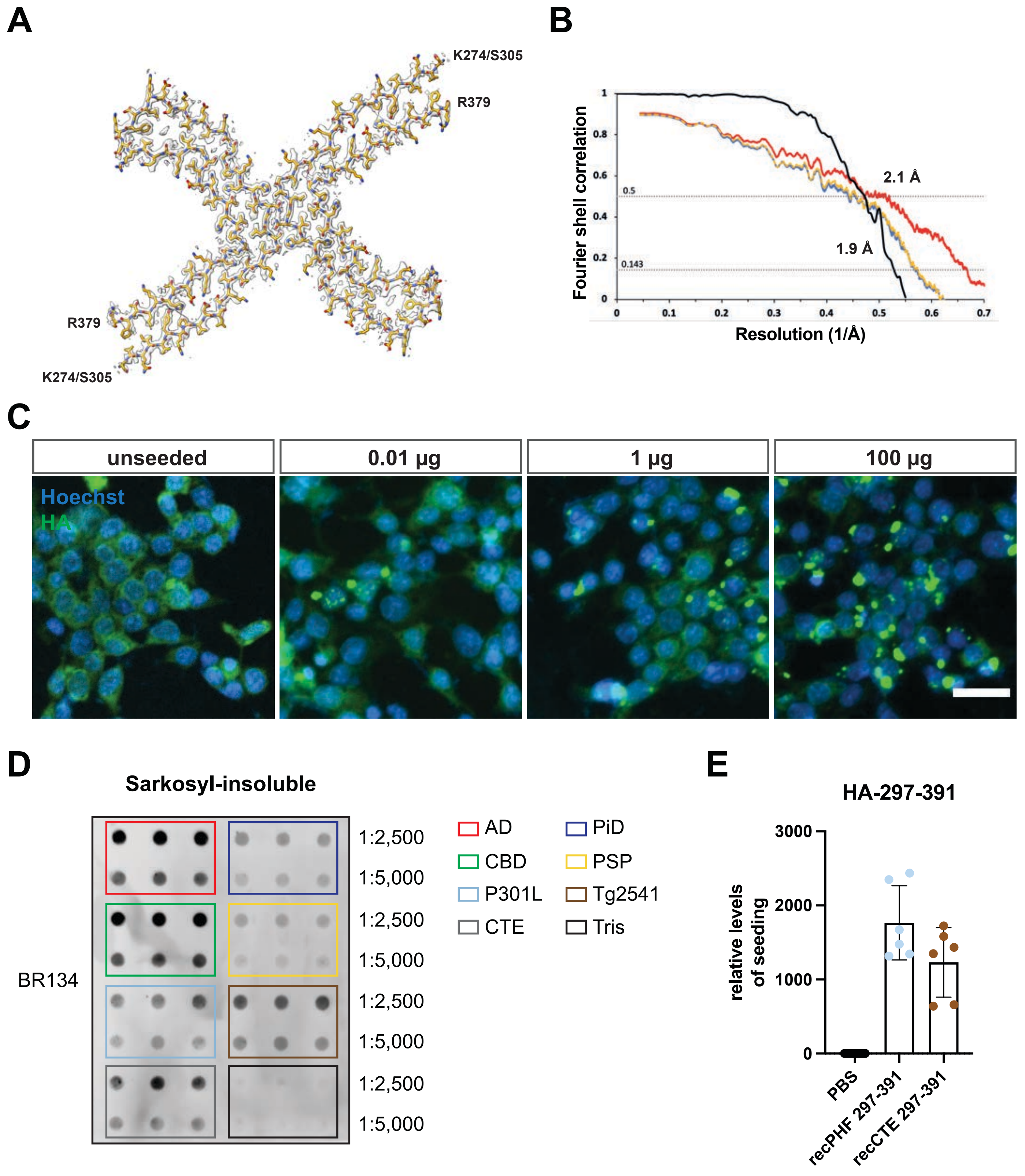

Figure S2. Cryo-EM structure determination of filaments with the CTE Type I fold, characterisation of brain-derived tau seeds and comparison of the seeding abilities of Alzheimer and chronic traumatic encephalopathy tau folds assembled from recombinant tau297-391

(A) Cryo-EM density map of the brain-derived sarkosyl-insoluble filaments with CTE Type I fold in transparent grey, superimposed by their refined atomic model. (B) Fourier shell correlation (FSC) curves for brain-derived CTE Type I filaments. Solvent-corrected FSC curves between independently refined half-maps are shown in black; FSC curves between the refined model and the reconstruction from all particles are shown in red; FSC curves between a model refined against half map 1 and that same half map are shown in dashed yellow; FSC curves between the same model and half map 2 are shown in blue. (C) Representative immunofluorescence images from HA-tau297-391 biosensors treated with increasing amounts of seeds extracted from AD brain. HA antibody and Hoechst dye were used. Scale bar, 35 μm. (D) Dot blot analysis with anti-tau antibody BR134 of sarkosyl-insoluble samples extracted from the brains of human tauopathy cases and from the brains of transgenic mice overexpressing human 0N4R tau P301S. Buffer only (Tris) was used as negative control. (E) Quantification of seeding in HA-tau297-391 biosensor cells with 500 ng of recombinantly assembled tau297-391 AD and CTE filaments. Image analysis included at least 13,800 cells per condition from three experimental replicates. Error bars denote standard deviations.

Supplementary figure 3

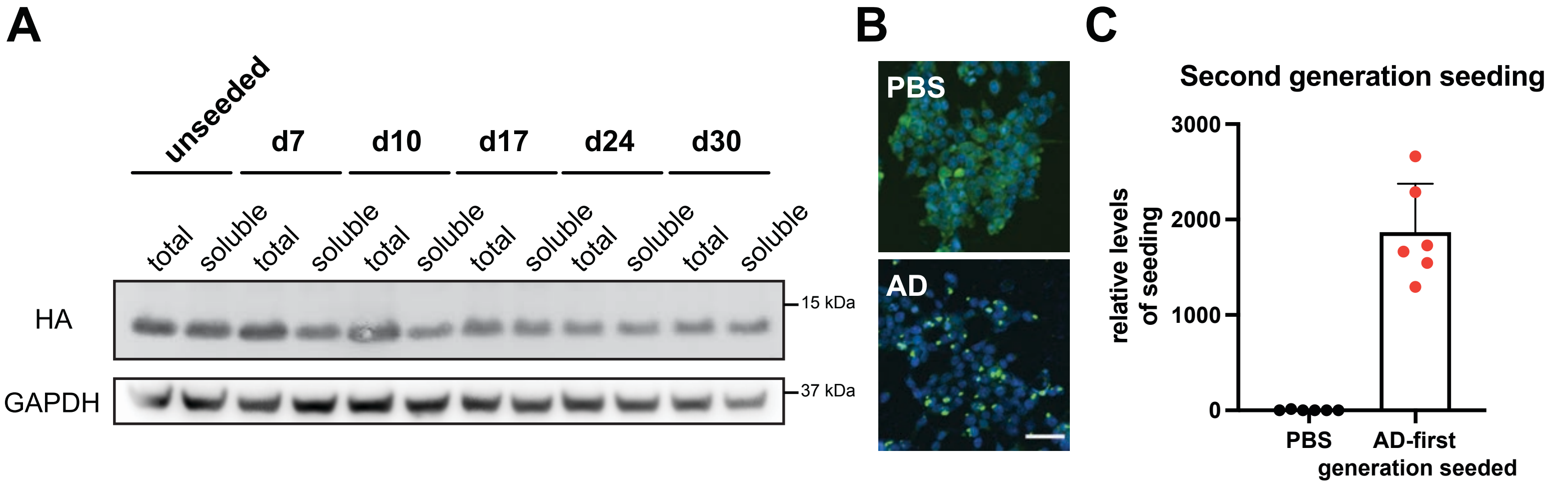

Figure S3. Western blot analysis of total cell lysate and soluble fractions at different times after seeding and second-generation seeding ability of Alzheimer-seeded HA-tau297-391 assemblies

(A) Time course Western blot analysis of total cell lysates and sarkosyl-soluble material extracted from HA-tau297-391 biosensors following seeding with AD-seeds. Anti-HA antibody was used to detect the HA-tau297-391 fragment, while GAPDH as loading control. (B) Representative immunofluorescence images of biosensor cells that were incubated with PBS or seeded with 1 µg of cell pellets from sarkosyl-extracted material of AD-seeded HA-tau297-391. HA antibody and Hoechst dye were used. Scale bar, 50 µm. (C) Quantification of second-generation seeding experiments with 1 µg of cell pellet from AD-seeded HA-tau297-391 biosensor cells. Image analysis included at least 2,400 cells per condition from three experimental replicates. Error bars denote standard deviations.

Supplementary figure 4

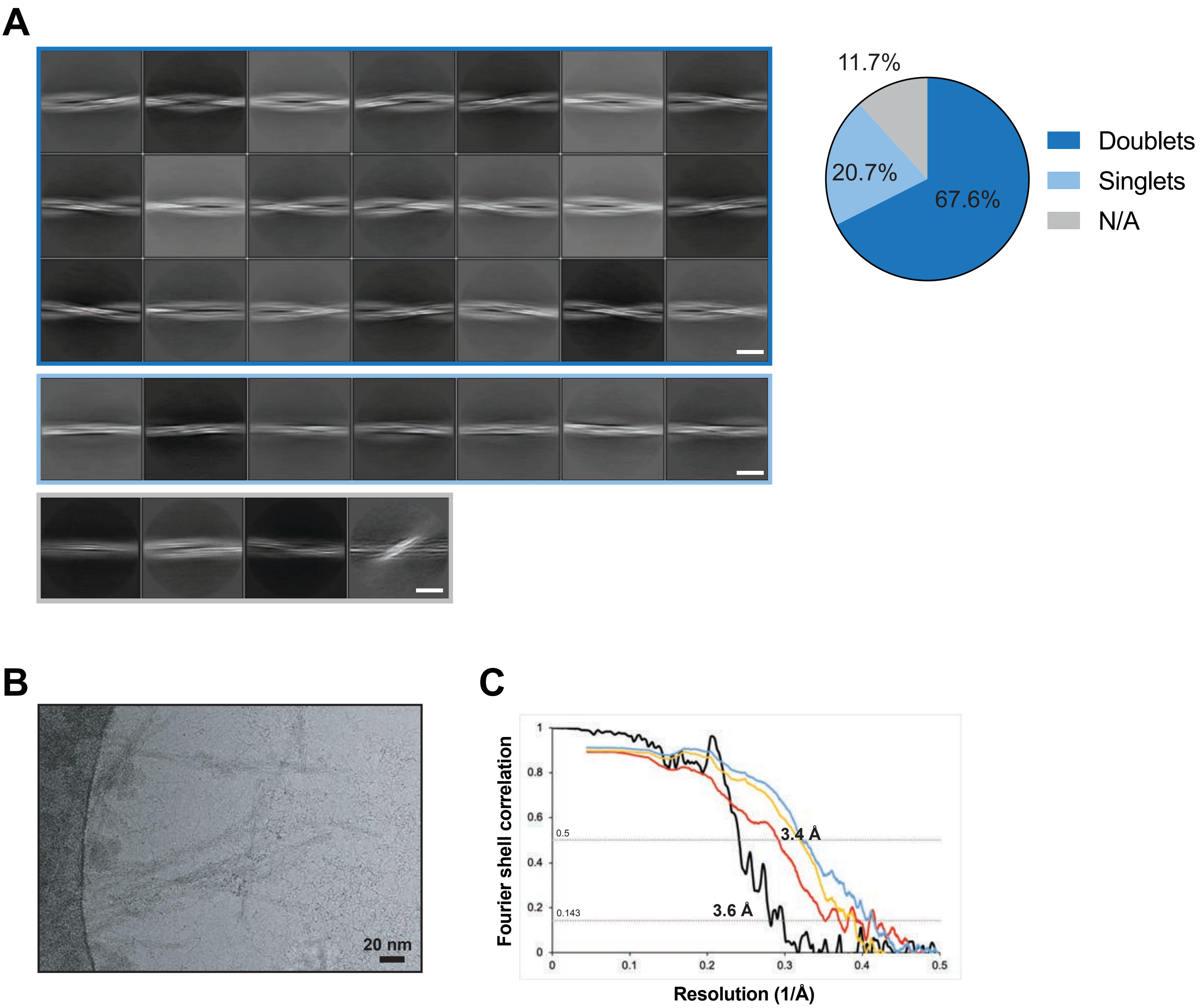

Figure S4. 2D-classification and cryo-EM structure determination of Alzheimer-seeded tau filaments

(A) Representative 2D-classification images of filaments from AD-seeded HA-tau297-391 biosensors and pie charts with the particle distribution per filament type (two protofilaments-doublets in dark blue; single protofilaments in light blue; discarded filaments in grey). Scale bars, 20 nm. (B) Cryo-EM micrograph from AD-seeded HA-tau297-391 filaments. Scale bar, 20 nm. (C) Fourier shell correlation (FSC) curves for HA-tau297-391 filaments from AD-seeded biosensors. Solvent-corrected FSC curves between independently refined half-maps are shown in black; FSC curves between the refined model and the reconstruction from all particles are shown in red; FSC curves between a model refined against half map 1 and that same half map are shown in dashed yellow; FSC curves between the same model and half map 2 are shown in blue.

Supplementary figure 5

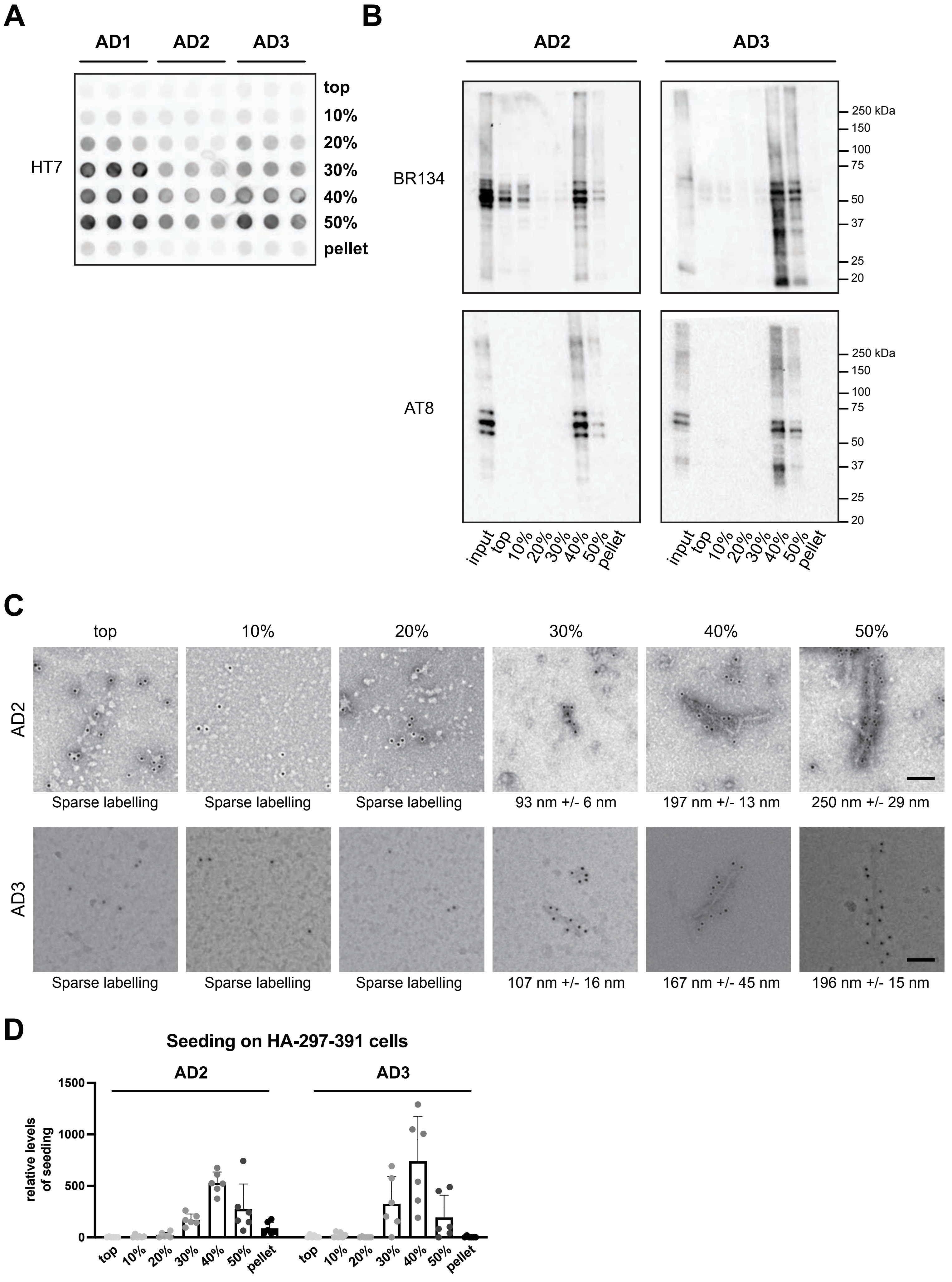

Figure S5. Tau species in the 40% sucrose fractions from the frontal cortex of Alzheimer's disease cases 2 and 3 are the most seed-competent

(A) Dot blot analysis of sucrose gradient fractions from AD cases 1, 2 and 3 following immunoblotting with anti-tau antibody HT7. (B) Western blot analysis with anti-tau antibodies BR134 and AT8 following SDS-PAGE of brain lysates fractionated by sucrose gradient centrifugation. (C) Immunoelectron microscopy of sucrose gradient fractions from AD cases 2 and 3 with BR134. The measured filament lengths (30%, 40%, and 50%) are shown ($n \geq$ at least 7 filaments measured per fraction) (D) Seeding ability of normalised sucrose gradient fractions from AD cases 2 and 3 in HA-tau297-391 biosensor cells. Image analysis included at least 2,000 cells per condition from three experimental replicates. Error bars denote standard deviations.

Supplementary figure 6

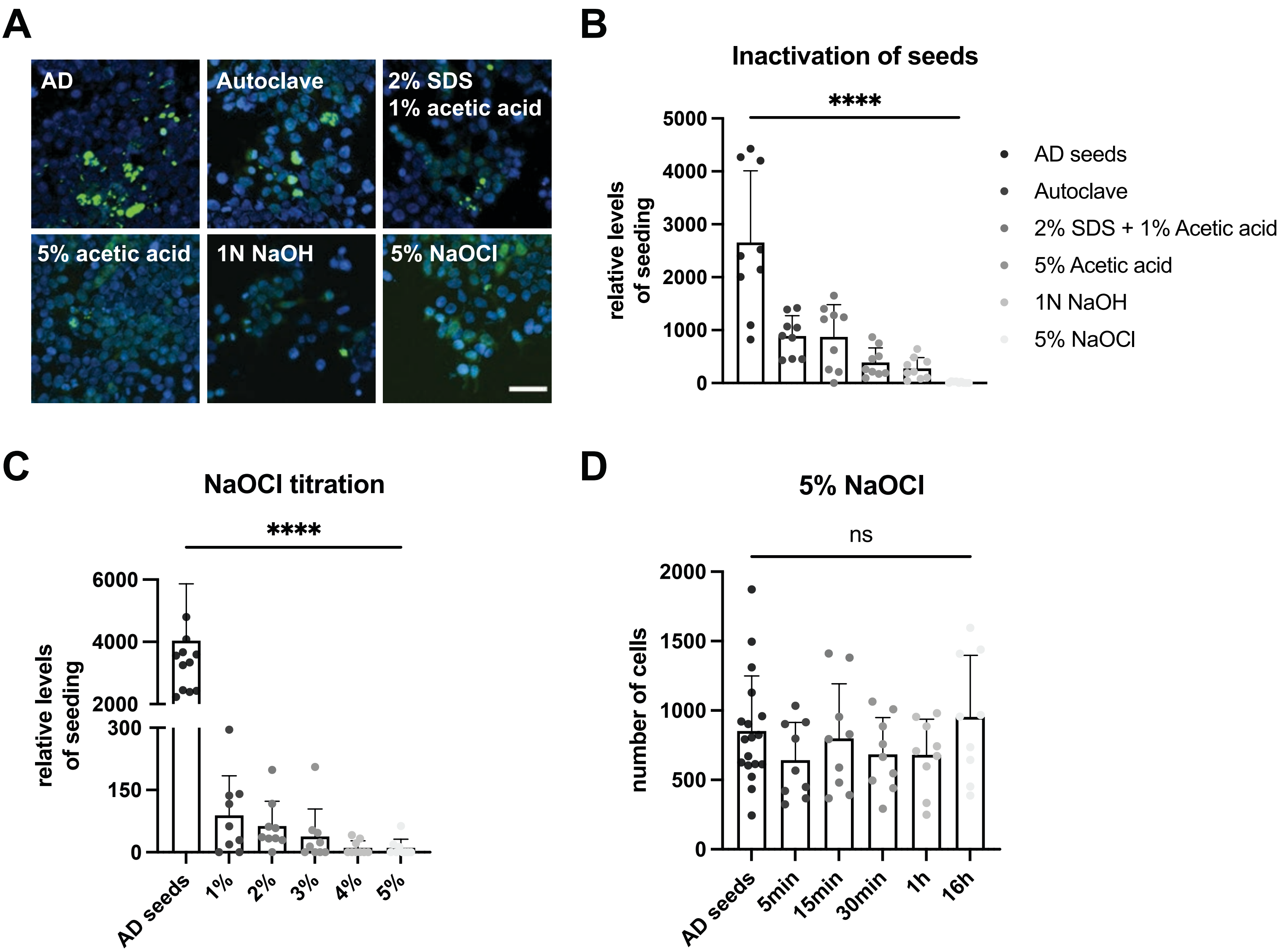

Figure S6. Inactivation of tau seeds extracted from Alzheimer's disease brain

Representative immunofluorescence images of HA-tau297-391 biosensor cells seeded with untreated or inactivated AD-derived tau seeds. Labelling with an anti-HA antibody and Hoechst dye. Scale bar, 50 µm. (B) Number of cells after seeding with AD-derived material treated with different inactivation methods. (C) Seeding potential of sarkosyl-insoluble material from AD after inactivation with increasing concentrations of NaOCl. (D) Biosensor cell numbers after addition of AD-derived seeds and treatment with 5% NaOCl. Image analysis included at least 2,000 cells per condition from three experimental replicates. Error bars denote standard deviations. $^{ns}P > 0.05$ and $^{****}P < 0.0001$ by one-way ANOVA with Tukey's correction in B, C, and D. Errors bars denote standard deviations.

Table S1. Cryo-EM data acquisition and model refinement statistics

	HA-297-391 sAD1 seeded EMD-54909 PDB 9SHS	CTE Type I
**Data collection and processing**		
Pixel size (Å)	0.93	0.46
Voltage (kV)	300	300
Detector	K3	Falcon 4i
Electron dose (e–/Å^2)	26.2	40.0
Energy filter slit (eV)	20	10
Defocus range (μm)	-1.0 to -2.0	-0.5 to -2.5
Initial particle images (no.)	1,000,773 (manual)	2,133,012 (Topaz)
Symmetry imposed	C2	C1
Final particle images (no.)	22,000	167,306
Map resolution (Å)	3.6	1.90
FSC threshold = 0.143		
Helical rise (Å)	4.83	2.40
Helical twist (°)	-0.93	179.417
**Model Refinement**		
Initial model used (PDB)	7QKI	6nwp
Model resolution (Å)	3.4	2.1
FSC threshold (Å) = 0.5		
Map sharpening *B* factor (Å^2)	-97.8	-45.8
Model composition		
Non-hydrogen atoms	3468	3444
Protein residues	456	450
Ligands	na	na
B factors (Å^2)		
Protein	71	115
Ligand	na	na
R.m.s. deviations		
Bond lengths (Å)	0.011	0.011
Bond angles (°)	1.978	1.879
Validation		
MolProbity score	1.07	0.64
Clashscore	0	0
Poor rotamers (%)	0	0
Ramachandran plot		
Favored (%)	89.19	97.26
Allowed (%)	9.23	2.74
Disallowed (%)	1.58	0
